# Molecular Link between Leaf Coloration and Gene Expression of Flavonoid and Carotenoid Biosynthesis in *Camellia sinensis* Cultivar ‘Huangjinya’

**DOI:** 10.3389/fpls.2017.00803

**Published:** 2017-05-24

**Authors:** Lubin Song, Qingping Ma, Zhongwei Zou, Kang Sun, Yuantao Yao, Jihan Tao, Najeeb A. Kaleri, Xinghui Li

**Affiliations:** ^1^Tea Research Center, Shandong Institute of PomologyTai’an, China; ^2^Tea Research Institute, College of Horticulture, Nanjing Agricultural UniversityNanjing, China; ^3^Department of Plant Science, University of Manitoba, WinnipegMB, Canada

**Keywords:** *Camellia sinensis* cv. Huangjinya, color changes, transcriptome, flavonoid biosynthesis, carotenoid biosynthesis

## Abstract

‘Huangjinya’ is an excellent albino tea germplasm cultivated in China because of its bright color and high amino acid content. It is light sensitive, with yellow leaves under intense light while green leaves under weak light. As well, the flavonoid and carotenoid levels increased after moderate shading treatment. However, the mechanism underlying this interesting phenomenon remains unclear. In this study, the transcriptome of ‘Huangjinya’ plants exposed to sunlight and shade were analyzed by high-throughput sequencing followed by *de novo* assembly. Shading ‘Huangjinya’ made its leaf color turn green. *De novo* assembly showed that the transcriptome of ‘Huangjinya’ leaves comprises of 127,253 unigenes, with an average length of 914 nt. Among the 81,128 functionally annotated unigenes, 207 differentially expressed genes were identified, including 110 up-regulated and 97 down-regulated genes under moderate shading compared to full light. Gene ontology (GO) indicated that the differentially expressed genes are mainly involved in protein and ion binding and oxidoreductase activity. Antioxidation-related pathways, including flavonoid and carotenoid biosynthesis, were highly enriched in these functions. Shading inhibited the expression of flavonoid biosynthesis-associated genes and induced carotenoid biosynthesis-related genes. This would suggest that decreased flavonoid biosynthetic gene expression coincides with increased flavonoids (e.g., catechin) content upon moderate shading, while carotenoid levels and biosynthetic gene expression are positively correlated in ‘Huangjinya.’ In conclusion, the leaf color changes in ‘Huangjinya’ are largely determined by the combined effects of flavonoid and carotenoid biosynthesis.

## Introduction

The tea plant [*Camellia sinensis* (L.) O. Kuntze] is an evergreen tree that is widely distributed in tropical and subtropical regions in the world. Tea is one of the most popular non-alcoholic beverages, and leaf color is an important indicator of tea quality (Taylor et al., 1992; Ravichandran, 2002). Catechins is a class of flavonoids and significantly correlated with chlorophyll content in tea (Wei et al., 2011). The catechins level is also affected by environmental factors especially light (Mariya et al., 2003; Premkumar et al., 2008).

Different environmental factors produce many variations in the leaf color of tea plant germplasms. However, some albino tea cultivars, such as the light-sensitive ‘Huangjinya,’ have exceptional qualities and a good taste (Li et al., 2016). Compared to normal green tea cultivars, the lower catechin levels of ‘Huangjinya’ make the tea taste less bitter (Feng et al., 2014). ‘Huangjinya’ has a different pigment composition than general tea cultivars: it has yellow leaves under intense light but green leaves under shade. However, the mechanism underlying the color changes in ‘Huangjinya’ is unclear.

Plant carotenoids are a family of pigments ranging in color from yellow to red that are involved in light harvesting and are indispensable for photoprotection against excess light. Carotenoids are synthesized in plastids such as chromoplasts and chloroplasts by various enzymes. Li et al. (2016) found that ‘Huangjinya’ leaves had higher amino acid and caffeine levels and lower polyphenol levels than tea cultivars with green leaves. They also suggested that 2-C-methyl-D-erythritol-4-phosphate (MEP) and carotenoid and chlorophyll metabolism-associated genes may contribute to the color changes in ‘Huangjinya.’ Nevertheless, whether the expression of these genes is the primary factor in color changes is still unclear.

It has also been reported that flavonoids participate in ultraviolet (UV) light-absorption (Xie et al., 2012), antioxidation and salinity stress tolerance (Agati et al., 2012; Ismail et al., 2015). The major flavonoids in tea plants are catechins, which are an important class of flavanols. Compared to green tea cultivars, ‘Huangjinya’ contains significant lower level of polyphenols or catechins (Feng et al., 2014; Li et al., 2016). In tea leaves, catechins is the main component of tea polyphenols. After moderate shading, the polyphenols content in ‘Huangjinya’ increased but remained less than that in green cultivars (Li et al., 2016). Catechins have been associated with antioxidant activity in plants (Škerget et al., 2005; Nakabayashi et al., 2014). However, the role flavonoid biosynthesis during the leaf color changes in ‘Huangjinya’ is unclear.

In the present study, we moderately shaded ‘Huangjinya’ plants for 10 days and the tea leaves turned from yellow to green colors. We therefore compared the phenotypes and *de novo* transcriptomes of ‘Huangjinya’ leaves grown in sunlight and in the shade to elucidate the mechanisms underlying the leaf color changes in ‘Huangjinya.’ We have also focused on the role of carotenoid and flavonoid biosynthesis played on the process of color changes in ‘Huangjinya.’

## Materials and Methods

### Plant Materials

Two-year-old tea plants growing well (*Camellia sinensis* cv. Huangjinya) in the experimental field of the Tea Institute of Shandong Academy of Agricultural Sciences in Tai’an, China (N36°11′, E117°10′, 153 m above sea level) were used in this experiment. One group of tea plants was shaded by a single layer shade net which reduced light intensity by about 45%, and another group of tea plants grown in the same field without the shade net was used as the control. The experiment was performed in late April with mean temperature of 26°C (day) and 13°C (night). After 10 days, at least 10 tender leaves at the third position from the top of both groups were harvested and frozen quickly in liquid nitrogen. Three biological repeats from different plants were performed for each group. These frozen leaves were stored at -80°C for further analysis.

### Measurement of Chlorophyll Content

The levels of chlorophyll and carotenoids, which are the major pigments influencing leaf color, were measured. Briefly, 100 mg of fresh tea leaves was ground by using a disposable pestle and then were extracted with 15 ml of 95% ethanol and incubated in the dark for 30 min. The extracts were filtered and analyzed with a UV-5800PC spectrophotometer (Metash, China). The absorbance at the following wavelengths was recorded: 665 nm for chlorophyll a, 649 nm for chlorophyll b, and 470 nm for total carotenoids. Total chlorophyll (Chl mg/g), carotenoid content and the Chl a/b ratio were calculated according to the method of Welburn and Lichtenthaler (1984).

### RNA Preparation and Sequencing

Total RNA was extracted using TRIzol (Vazyme Biotech, Nanjing, China). Briefly, 0.1 g tea leaves was ground in 1.5 ml Trizol by homogenizer (Scientz Biotech, Ningbo, China) and centrifuged for 5 min, 1/5 volume chloroform was added in the supernatant followed by centrifugation for 15 min. Thereafter, the equal volume isopropanol was added in supernatant for precipitating RNA. Finally, the precipitation was washed by 75% ethanol for three times and dissolved in 100 μl diethylpyrocarbonate treated water. RNA integrity and concentration was detected by 1.2% agarose gel electrophoresis and Agilent 2100 Bioanalyzer. cDNA libraries were obtained using the VAHTS^TM^ Stranded mRNA-seq Library Prep Kit for Illumina^®^ by Vazyme Biotech and were then sequenced using the Illumina HiSeq 4000 platform by Vazyme Biotech. All the samples were sequenced on the same lane.

### *De Novo* Assembly

The raw data were filtered by removing the adapter sequences, low-quality reads (the rate of reads which quality value ≤ 10 is more than 20%) and the reads with unknown nucleotides larger than 5%, and clean reads were produced for further analysis. *De novo* transcriptome assembly was carried out using the Trinity program as described previously (Grabherr et al., 2011). The assembled unigenes were analyzed with the TGI Clustering Tool (TGICL) (Pertea et al., 2003) to remove redundant sequences and then clustered with the Phrap assembler^1^ to obtain distinct sequences. The fragments per kb per million fragments (FPKM) method was used to calculate the expression of unigenes (Audic and Claverie, 1997). The differentially expressed genes (DEGs) were screened by R software using DESeq package (Anders and Huber, 2010). False Discovery Rate (FDR) ≤ 0.001 and |log2Ratio|≥ 1 was considered as the threshold of the differential expression.

### Annotation and CD Prediction

The GO classifications of unigenes were analyzed by Blast2Go software with default parameters (Conesa et al., 2005). To understand the categorization of gene functions of the tea leaves, Web Gene Ontology Annotation Plot (WEGO) software (Ye et al., 2006) was used to conduct GO functional classification thereafter. All of the unigenes were aligned to the Non-redundant protein sequences (NR), Swiss-Prot, Kyoto Encyclopedia of Genes and Genomes (KEGG), and Cluster of Orthologous Groups of proteins (COG) databases using Blastx, and nucleotide sequences database (NT) using Blastn with *e*-values < 1.0e^-5^.

For predicting coding regions (CDs), NR, Swiss-Prot, KEGG, COG were blasted in order with *e*-value < 1.0e^-5^. Unigenes that could not be aligned to any database were further scanned with ESTScan (Iseli et al., 1999).

### Sequence Alignment and Phylogenetic Tree Construction

In order to understand the structure of a novel flavonoid-related gene *senescence-related gene 1* (*SRG1*, CL6306.Contig1), the protein sequences of SRG1 and flavonol synthase (FLS) from different plants were aligned by CLUSTALW online^2^ with the default parameters and the phylogenetic tree was constructed by Mega 7.0 using the Neighbor-Joining method with 1000 bootstrap replications (Kumar et al., 2016). The evolutionary distances were computed using the Poisson correction method. The phylogenetic tree was rooted with the outgroup of flavanone-3-hydroxylase (F3H) protein sequences from *Arabidopsis thaliana, Chrysanthemum* × *morifolium, C. sinensis*, and *Vitis rotundifolia*. The motifs of the SRG1 sequences were predicted by Multiple Em for Motif Elicitation (MEME) Version 4.11.2 online^3^.

### Quantitative Real-Time PCR Analysis

A total of 1 μg of extracted total RNA was used to synthesize first-strand cDNA with the RevertAid^TM^ First Strand cDNA Synthesis Kit (Fermentas, Canada) as described in the manual. QRT-PCR analysis was performed in a 25 μl volume containing 9.5 μl ddH_2_O, 1 μl primers, 2 μl cDNA, and 12.5 μl SYBR Premix EX Taq (Takara, Japan). The qRT-PCR primers were listed in **Table 1** and Supplementary Table S1. The *GAPDH* (GenBank: GE651107) of tea plant was used as the reference gene. The primers are F: TTGGCATCGTTGAGGGTCT and R: CAGTGGGAACACGGAAAGC. The following procedure was used on a CFX96 Real-Time PCR Detection System (BioRad, USA): 95°C for 30 s, followed by 40 cycles of 95°C for 5 s and 60°C for 30 s. Relative expression was calculated with the 2^-ΔΔC_t_^ method (Livak and Schmittgen, 2001). The statistical analysis was performed using Excel 2010 and SigmaPlot 13.0 (Systat Software, Canada). Student *t*-test was used to evaluate the difference between two samples and *P* < 0.05 was considered significant statistically. Each of the two samples has three biological replicates.

**Table 1:** The annotation of and primers for the genes verified by qRT-PCR.

GO term	Gene ID	Gene annotation	Forward primer (5′-3′)	Reverse primer (5′-3′)	Log2 Ratio
Protein binding	CL3897.Contig4	Heat shock protein 90 (HSP90)	GCGAGAATCTTGGTAGGGGTAC	CTTCCTCATCCTCATCATCAG	3.56
	Unigene52222	HSP90-2	CAAGCAAGAAGACCATGGAGAT	TCCTCGTTAGCCTCCTCTTCC	2.86
Ion binding	Unigene46400	Rubredoxin-like superfamily protein (RLS)	TCTATCATCTCCCACCACCG	CCACAAACAGGACAGAAGTAGC	–1.48
	Unigene29199	Inositol-pentakisphosphate 2-kinase-like (IPK)	ACTCTATTGTCCTTGACCCG	TACCAAACATCGCATCTCTG	–1.99
	Unigene21794	Probable BOI-related E3 ubiquitin-protein ligase (BOI)	TGATGGACGATGCTCAGTC	CGACGACCCACAAACAGTAC	1.54
Oxidoreductase activity	Unigene2456	Luminal-binding protein 5 (LBP)	TGATGACCAAGTTGATTCCTAGG	CCTTGTCTTCAGCCTTCACAT	1.82
	Unigene16685	Gibberellin 2-beta-dioxygenase 8 (G2D8)	TCTACATCGACTGGTAACCTG	GCATCATATCACCGAGGAGAAG	–1.68
	Unigene39616	Hsp70-2	CAACATGAGGAATACAGTCAAG	GCAGTAGGAACATCATCGTCAT	2.14
	Unigene40387	Hsp70-15	TGAATGTGGGCAAGCTGAAG	GCTCCAGCATTCTCGTTAGG	1.59
	CL4428.Contig1	Receptor-like protein kinase HAIKU2-like (RPKH)	GTCTTTAGGCTCGTGTGTTTC	CACCGACAAATGCTTCAATC	1.76
	CL6306.Contig1	Senescence-related gene 1 (SRG1)	TTGTTTGAAGAAGGGATGCAG	GGCTTCAGAGAGGGGTTTAAC	3.48
	CL6544.Contig1	Cytochrome P450 85A	CATGACCACCCTGATGTTCTTC	CGGGTTGAAGGTTAATGGGTTAG	2.49
	CL819.Contig2	Linoleate 13S-lipoxygenase 2-1 (LO)	TGATGGTCTTGTTCTCTGGGATG	GAGTGGTGACCGGAGGCTAC	2.05
	CL8169.Contig1	Flavonol synthase (FLS)	GCATGAGGTCAAGGAGGCTGT	GACAATCAGGGCATTAGGGATG	–2.02
	Unigene28864	Flavonoid-3′-hydroxylase (F3’H)	TCGAATGGCATCTGACAGTTG	GCCTGCACCAAATGGTATGAC	–1.88

## Results

### Phenotypic Changes in the Tea Plants after Shading

**FIGURE 1:**
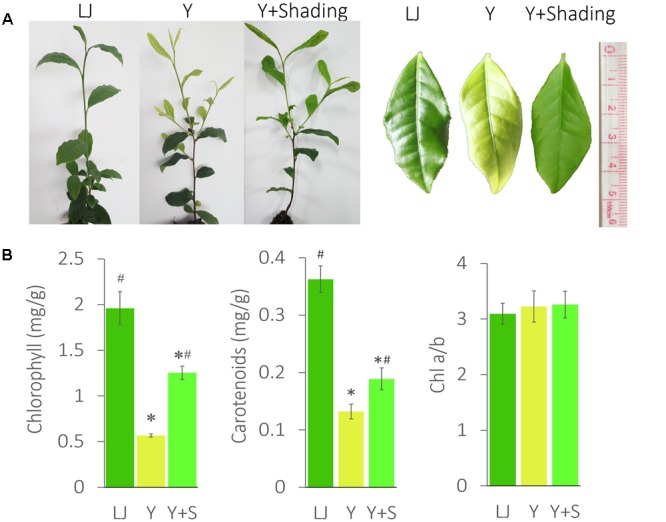
**The phenotype and pigment changes in ‘Huangjinya.’ (A)** The leaf color changes of ‘Huangjinya’ after shading. **(B)** The changes in chlorophyll and carotenoid content. LJ is the green tea cultivar ‘Longjingchangye,’ Y is ‘Huangjinya’ without shading, and Y+S is ‘Huangjinya’ with shading. ^∗^ indicates a significant difference compared to LJ (*P* < 0.05); ^#^ indicates a significant difference (*P* < 0.05) compared to Y. The data are presented as the mean ± standard deviation (*n* = 10).

‘Huangjinya’ (Y) showed a significantly lighter leaf color and lower chlorophyll content than ‘Longjingchangye’ (LJ), a tea cultivar with green leaves (**Figure 1**).

The shaded ‘Huangjinya’ leaves (Y+Shading, Y+S) turned green within 7 days and were analyzed after 10 days. Chlorophyll content in shaded leaves clearly increased but was still lower than that in LJ plants. The carotenoid content changed in a manner similar to that observed for chlorophyll. Nevertheless, no significant difference was found in Chl a/b among the tea plants, indicating consistent photosynthetic performance in these tea plants.

### *De Novo* Assembly

**FIGURE 2:**
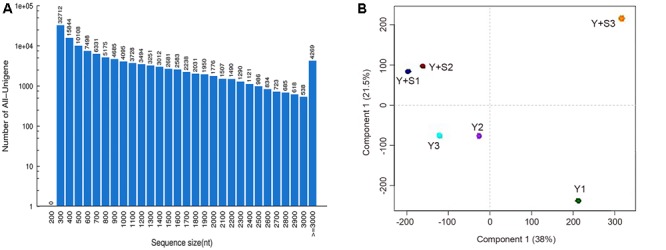
**The sequence sizes of all the unigenes (A)** and the correlation analysis of the samples **(B)**. Y+S1-3 and Y1-3 are three replicates of tea plants with and without shading, respectively.

In order to explain the mechanism of color changes of ‘Huangjinya,’ the transcriptomes of ‘Huangjinya’ during sunlight and shading treatment were compared. A total of 32.5 billion nt of raw data were generated from Illumina HiSeq 4000 sequencing. All of the samples were high quality, with a minimum Q20 of 94.65% (**Table 2**). *De novo* assembly revealed 127,253 unigenes with a total length of 116.3 million nt, an average length of 914 nt and an N50 of 1,510 nt (**Table 3**). The length distribution of all of the unigenes was shown in **Figure 2A**. The assembly unigenes and expression data have been submitted to Gene expression Omnibus^4^ with accession number GSE97659.

**Table 2:** Quality assessment of the raw data.

Samples	Accession No.	Total raw reads	Total clean reads	Total clean nucleotides (nt)	Q20 (%)	*N* (%)	GC Content (%)
Y+S1	SRR5075641	78,123,142	64,311,466	8,038,933,250	96.22	0.01	45.72
Y+S2	SRR5075642	59,027,752	47,616,458	5,952,057,250	96.50	0.01	45.25
Y+S3	SRR5075643	44,890,354	36,234,136	4,529,267,000	94.65	0.01	45.75
Y1	SRR5074626	50,220,132	40,990,016	5,123,752,000	96.34	0.01	45.22
Y2	SRR5075639	44,323,668	36,635,114	4,579,389,250	94.64	0.01	45.57
Y3	SRR5075640	42,309,104	34,359,304	4,294,913,000	96.28	0.01	45.18

*Y+S1-3 and Y1-3 are three replicates of tea plants with and without shading, respectively.*

**Table 3:** Quality of the assembled unigenes.

Sample	Total number	Total length (nt)	Mean length (nt)	N50	Total consensus sequences	Distinct clusters	Distinct singletons
Y+S1	123,322	82,510,322	669	1306	123,322	38,578	84,744
Y+S2	125,145	78,428,448	627	1190	125,145	36,340	88,805
Y+S3	85,683	44,032,332	514	815	85,683	20,660	65,023
Y1	88,744	44,937,977	506	795	88,744	24,619	64,125
Y2	93,928	55,303,430	589	1046	93,928	28,192	65,736
Y3	102,455	61,791,465	603	1100	102,455	31,002	71,453
All	127,253	116,296,242	914	1510	127,253	47,390	79,863

*Y+S1-3 and Y1-3 are three replicates of tea plants with and without shading, respectively.*

Principal component analysis (PCA) revealed that Y+S3 in the shaded group (with correlation coefficients of 0.71 and 0.51 compared to Y+S1 and Y+S2, respectively) and Y1 in the control group (with correlation coefficients of 0.86 and 0.85 compared to Y2 and Y3, respectively) deviated from the other two replicates (**Figure 2B**). Therefore, these two samples were removed when predicting DEGs. The high correlation coefficients of 0.89 (Y+S1 vs. Y+S2) and 0.99 (Y2 vs. Y3) indicated that other two samples in each group could be integrated in the search for DEGs.

### Functional Annotation of the Tea Leaf Transcriptome

**FIGURE 3:**
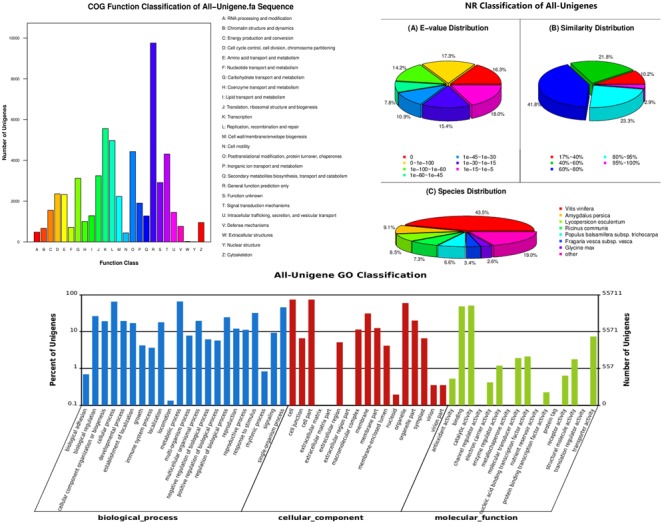
**Functional annotation and classification of all the unigenes identified in the tea plant, as determined by the Cluster of Orthologous Groups (COG), Non-redundant (NR), and gene ontology (GO) databases**.

We obtained 81,128 annotated genes for functional annotation analysis, including 74,923, 66,959, 48,987, 45,566, 29,478, and 55,711 unigenes annotated by the NR, NT, Swiss-Prot, KEGG, COG, and GO databases, respectively. As shown in **Figure 3**, most of the predicted genes were mapped to “general function prediction only,” “transcription” and “replication, recombination, and repair” in the COG functional classification analysis. NR classification analysis showed that tea transcriptome had the highest similarity to that of *Vitis vinifera* (43.5%). GO classification revealed that more than half biological processes gathered 10% unigenes. GO cellular component classification indicated that cell part, membrane and organelle enriched the most unigenes in “Huangjinya.” In addition, most of the unigenes were found to act on binding and catalytic activity from the GO function analysis.

For protein CD prediction analysis, a total of 81,573 proteins were identified, including 75,070 CDs from the protein databases by blastx and 6,503 predicted CDs from ESTScan analysis.

### Gene Ontology and KEGG Analyses of Differentially Expressed Genes

**FIGURE 4:**
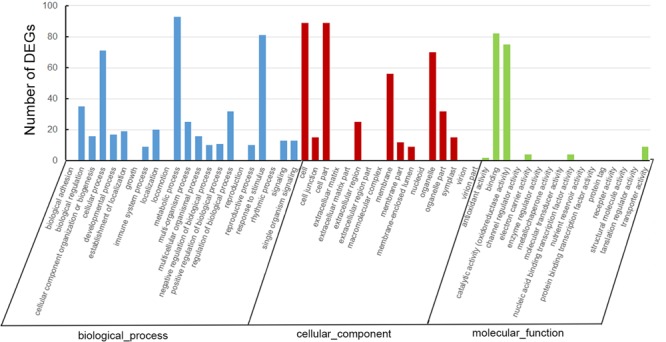
**Gene ontology analysis of differentially expressed genes (DEGs) in ‘Huangjinya’ after shading treatment**.

A total of 207 DEGs, including 110 up-regulated and 97 down-regulated genes, were identified in the tea transcriptome after shading (Supplementary Table S2). As shown in **Figure 4**, GO analysis indicated that most of the DEGs were involved in the cellular process, metabolic process and response to stimulus. GO cellular component analysis revealed the cell part, membrane and organelle enriched the most DEGs. Meanwhile, GO function analysis showed that the DEGs contributed to binding and catalytic activity (oxidoreductase activity).

**FIGURE 5:**
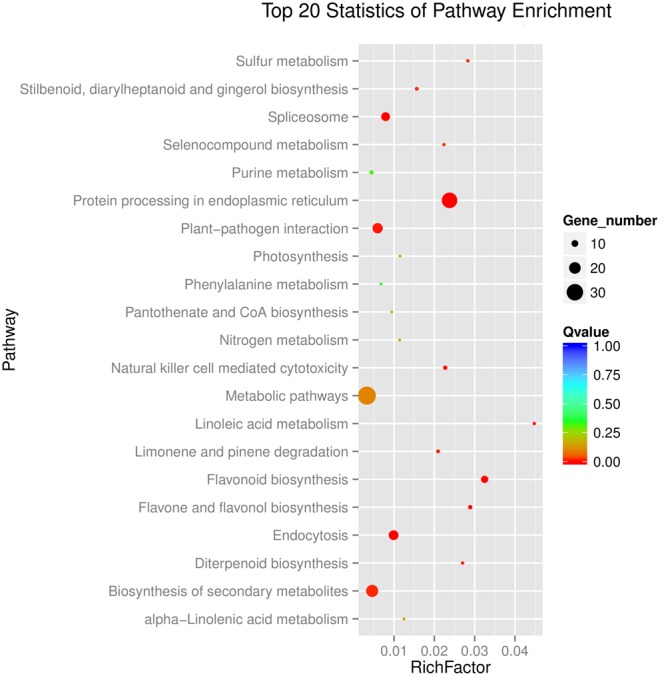
**Functional distribution of the DEGs in the tea plant transcriptome**. The enrichment factor is the ratio between DEGs in the pathway and all the annotated genes in the tea transcriptome. A large enrichment factor denotes a high degree of enrichment. The lower the *Q*-value is, the more significant the enrichment of the DEG.

**FIGURE 6:**
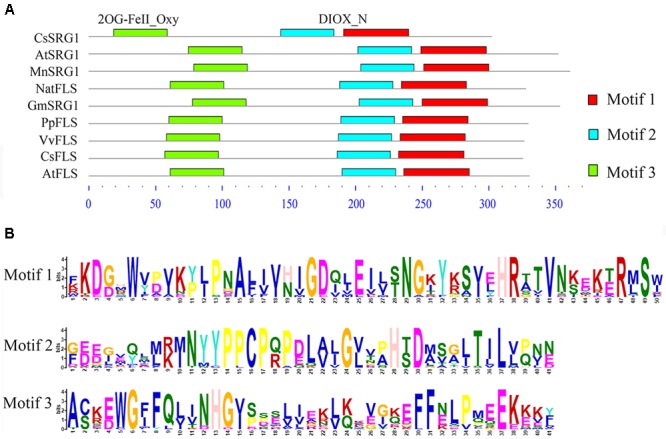
**The motif analysis of senescence-related gene 1 (SRG1) and flavonol synthase (FLS) proteins from different plants. (A)** The sequence alignment of SRG1 and FLS proteins. *Camellia sinensis* (CsSRG1, CL6306.Contig1; CsFLS, ABM88786.1), *Arabidopsis thaliana* (AtSRG1, Q39224.1; NP_001190266.1), *Morus notabilis* (MnSRG1, XM_010096827.1), *Glycine max* (GmSRG1, XM_003540470.3), *Narcissus tazetta* (NatFLS, AFS63899.1), *Prunus persica* (PpFLS, AJO70134.1), *Vitis vinifera* (VvFLS, BAE75810.1). **(B)** Motif sequences of the SRG1 and FLS proteins.

**FIGURE 7:**
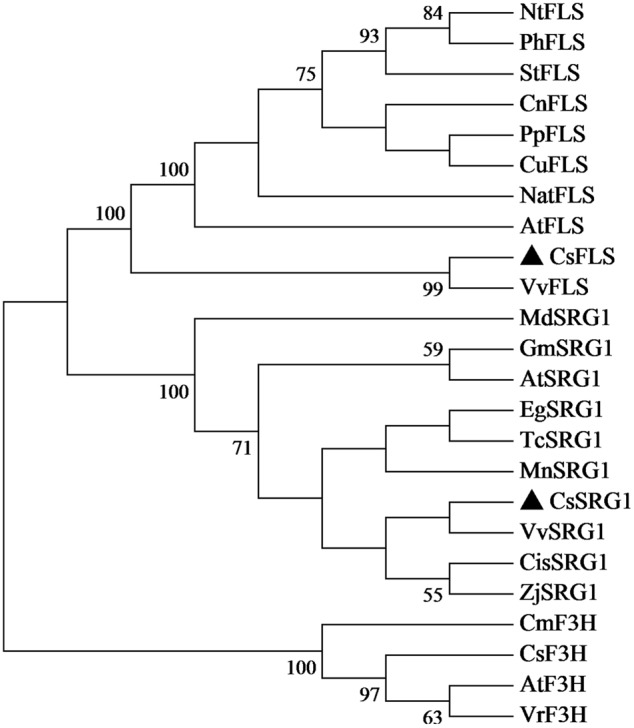
**Phylogenetic tree of SRG1 and FLS genes.** *Camellia sinensis* (CsSRG1, CL6306.Contig1; CsFLS, ABM88786.1; CsF3H, AAT68774.1), *Eucalyptus grandis* (EgSRG1, XM_018872917.1), *Theobroma cacao* (TcSRG1, XM_018123779.1), *Vitis vinifera* (VvSRG1, XM_002269051.2; VvFLS, BAE75810.1), *Morus notabilis* (MnSRG1, XM_010096827.1), *Citrus sinensis* (CisSRG1, XM_006489576.2), *Ziziphus jujuba* (ZjSRG1, XM_016021136.1), *Glycine max* (GmSRG1, XM_003540470.3), *Arabidopsis thaliana* (AtSRG1, Q39224.1; AtFLS, NP_001190266.1; AtF3H, NP_190692.1), *Malus domestica* (MdSRG1, XM_017335001.1), *Narcissus tazetta* (NatFLS, AFS63899.1), *Nicotiana tabacum* (NtFLS, ABE28017.1), *Petunia hybrida* (PhFLS, CAA80264.1), *Solanum tuberosum* (StFLS, CAA63092.1), *Camellia nitidissima* (CnFLS, ADZ28516.1), *Prunus persica* (PpFLS, AJO70134.1), *Citrus unshiu* (CuFLS, Q9ZWQ9.1), *Chrysanthemum × morifolium* (CmF3H, AEP37359.1), *Vitis rotundifolia* (VrF3H, AGS57503.1).

Kyoto Encyclopedia of Genes and Genomes enrichment analysis showed that these genes were involved in various metabolic pathways in tea plants (**Figure 5**). Most of the DEGs were enriched in protein processing (25.69%) and metabolism (30.28%). Flavonoid biosynthesis and linoleic acid metabolism pathways showed the highest enrichment factors. In addition, few DEGs were enriched in the photosynthesis pathway. Considering flavonoid-richness of tea and the color of ‘Huangjinya,’ we paid more attention to the metabolic pathways especially flavonoid and carotenoid biosynthesis. Besides *FLS* (CL8169.Contig2), another 2-oxoglutarate-dependent dioxygenase gene (SRG1, CL6306.Contig1) was differentially expressed and placed into the KEGG category flavonoid biosynthesis. Both SRG1 and FLS proteins contained two specific domains of DIOX_N and 2OG-FeII_Oxy which were conserved in 2OG-Fe(II) oxygenase superfamily (**Figure 6** and Supplementary Figure S1). The phylogenetic tree showed that SRG1 proteins were classified into the same cluster, but deviated from all the FLS proteins, indicating that the SRG1 protein in tea plant is separate from FLS (**Figure 7**). Therefore, we suggest that SRG1 and FLS are different proteins but paralogs.

### Relative Expression of the Differentially Expressed Genes

**FIGURE 8:**
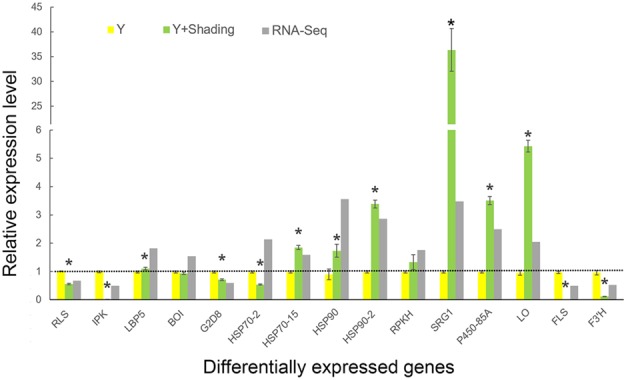
**Comparison of RNA-Seq data and quantitative RT-PCR verification of the DEGs.** Yellow and green bars represent expression of DEGs based on qRT-PCR data. Y indicates ‘Huangjinya’ exposed to sunlight. Gray bars represent fold change of DEG expression levels after shading based on RNA-Seq data. DEG expression levels under sunlight from RNA-Seq data were considered as 1, which were displayed as the dotted line. The qRT-PCR data were presented as the mean ± standard deviation (*n* = 3). ^∗^ means expressed significantly compared to Y (*P* < 0.05).

QRT-PCR was used for verification of the RNA-Seq results and samples used for qRT-PCR verification were independent of that for RNA-Seq analysis. Fifteen DEGs were selected for qRT-PCR verification (**Figure 8**). Of these genes, 2 were involved in ATP binding, 3 were associated with ion binding, and 10 were encoded proteins with oxidoreductase activity. The expression profile of most of the selected genes was similar in the qRT-PCR and RNA-Seq analyses, demonstrating that the RNA-Seq data was reliable.

**FIGURE 9:**
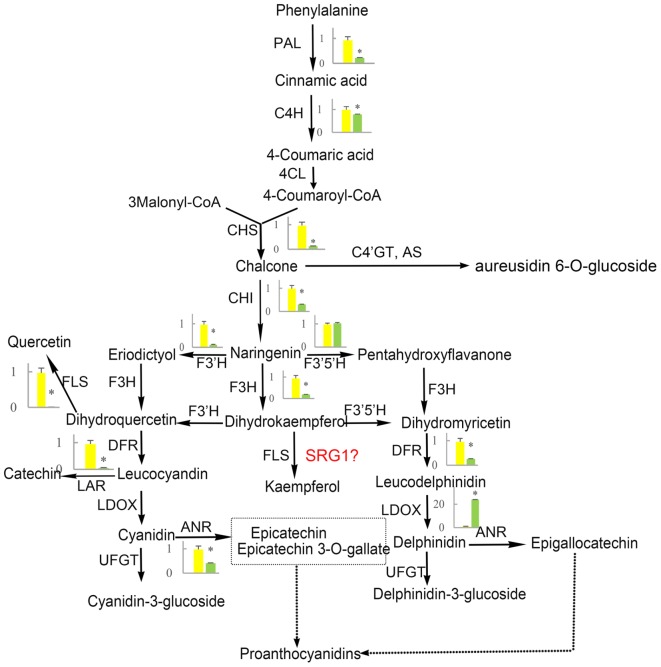
**The effect of shading on the expression of flavonoid synthesis-related genes in ‘Huangjinya.’** PAL, phenylalanine ammonia-lyase; C4H, cinnamate 4-hydroxylase; 4CL, 4-coumarate: CoA-ligase; CHS, chalcone synthase; AS, aureusidin synthase; C4′GT, chalcone 4′-*O*-glucosyltransferase; CHI, chalcone isomerase; F3H, flavanone-3-hydroxylase; F3′H, flavonoid 3′-hydroxylase; F3′5′H, flavonoid 3′,5′-hydroxylase; FLS, flavonol synthase; DFR, dihydroflavonol 4-reductase; LDOX, leucoanthocyanidin dioxygenase; UFGT, UDP-glucose:flavonoid 3-*O*-glucosyltransferase; LAR, leucoanthocyanidin reductase; ANR, anthocyanidin reductase. Green and yellow represent the gene expression in ‘Huangjinya’ with and without shading, respectively. ^∗^ indicates a significant difference (*P* < 0.05).

**FIGURE 10:**
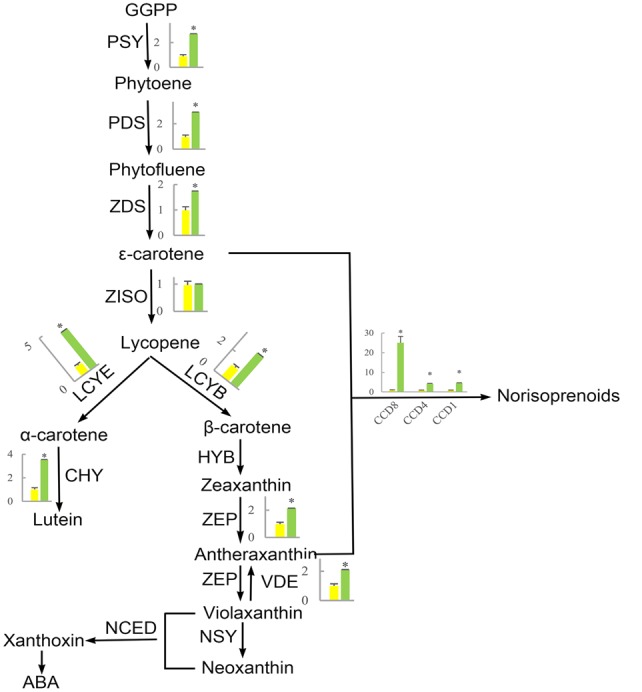
**The effect of shading on the expression of carotenoid synthesis-related genes in ‘Huangjinya.’** PSY, phytoene synthase; PDS, phytoene desaturase; ZDS, ζ-carotene desaturase; ZISO, ζ-carotene isomerase; LCYE, lycopene 𝜀-cyclase; LCYB, lycopene β-cyclase; CHY, carotenoid hydroxylase; HYB, β-ring hydroxylase; ZEP, zeaxanthin epoxidase; VDE, violaxanthin de-epoxidase; NSY, neoxanthin synthase; NCED, 9-cis-epoxycarotenoid dioxygenase; CCD, carotenoid cleavage dioxygenase. Green and yellow bars represent the gene expression of ‘Huangjinya’ with and without shading, respectively. ^∗^ indicates a significant difference (*P* < 0.05).

Flavonoid and carotenoid biosynthetic metabolisms are two important pathways associated with oxidoreductase activity. The expression of all genes associated with flavonoid and carotenoid biosynthesis were evaluated except for DEGs identified by RNA-Seq analysis (CL8169.Contig1, CL8169.Contig1, Unigene28864). QRT-PCR analysis showed that, with the exception of the *leucoanthocyanidin dioxygenase gene* (*LODX*), the genes involved in flavonoid synthesis were down-regulated in shading (**Figure 9**). In contrast, the genes associated with carotenoid biosynthesis were up-regulated in shading (**Figure 10**). These genes were not differentially expressed in RNA-Seq analysis may be due to the strict parameter setting of DEGs screening.

## Discussion

In this study, the transcriptome of a light-induced albino tea cultivar ‘Huangjinya’ was analyzed by RNA-Seq to explore the mechanisms responsible for the color changes in its leaves. Two hundred and seven genes were differentially expressed in shaded plants and plants grown under natural light. Data mining of comparative transcriptomes revealed that these DEGs are mainly involved in oxidoreductase activity and binding. KEGG pathway analysis indicated that metabolic pathways, including the carotenoid and flavonoid biosynthetic pathways, play important roles in the color change mechanisms of ‘Huangjinya.’

### Flavonoid Biosynthesis is Responsible for Light-Induced Color Changes in Tea Leaves

Flavonoid biosynthesis is catalyzed by a series of enzymes, including oxoglutarate-dependent dioxygenases (F3H, FLS, and LDOX), NADPH-dependent reductases [dihydroflavonol 4-reductase (DFR)], and cytochrome P450 hydroxylases [flavonoid 3′-hydroxylase (F3′H) and flavonoid 3′,5′-hydroxylase (F3′5′H), **Figure 9**]. Our results indicated that, with the exception of *F3*′*5*′*H* and *LDOX*, all of the genes in the flavonoid biosynthesis pathway were down-regulated by shading. Meanwhile, the suppression of these genes promoted the production of catechins. This is not the first report about the negative correlation between catechin content and expression of flavonoid biosynthesis-related genes. The same result has been reported in another albino tea cultivar ‘Anjibaicha’ which showed that the expression of *PAL* controlling the first step of flavonoid biosynthesis was negatively associated with catechin (*r* = -0.683) or total polyphenol accumulation (*r* = -0.717) (Xiong et al., 2013). In addition, in a normal green tea cultivar ‘Shuchazao,’ the expression of flavonoid biosynthesis-related genes was also supressed by shading (Wang et al., 2012). However, there is a positive correlation between catechin content and expression of flavonoid biosynthesis-related genes in ‘Shuchazao.’ Nevertheless, in ‘Huangjinya,’ the expression of flavonoid biosynthesis associated genes was inhibited but polyphenols (whose major constitutes are catechins) were elevated after moderate shading (Li et al., 2016). However, tea polyphenols decreased under strong shading, suggesting tea polyphenols may be affected significantly by degree of shading. In our experiment, ‘Huangjinya’ were just shaded by a single layer shade net with about 45% decrease of light intensity, which conformed with the moderate shading by Li et al. (2016). As for increase of polyphenols under strong shading, it may be considered to be a possible stress induced by the light which is too weak to afford normal activities of plants.

It has been proved that chalcone and aureusidin are yellow pigments and contribute to the coloration of yellow flowers (Nakayama et al., 2000; Ono et al., 2006). In carnation, mutation of *chalcone isomerase (CHI)* and *DFR* led to the accumulation of chalcone 2′-glucoside and produced yellow flowers (Itoh et al., 2002). As well, knockdown of endogenous *DFR* and *F3H* from Antirrhinum would block anthocyanin biosynthesis and result in yellow flowers. In tea plants, yellow leaves of ‘Huangjinya’ showed obvious lower total catechins (precursor for biosynthesis anthocyanin) than green tea leaves (Feng et al., 2014), suggesting that the catechins biosynthesis was inhibited. Given that the negative correlation of polyphenols and the expression flavonoid biosynthesis-related genes in ‘Huangjinya,’ we surmise that light-induced expression of flavonoid biosynthesis-related genes would suppress the accumulation of polyphenols. This may be a part of the reason for the light color of ‘Huangjinya’ plants grown in sunlight.

Another cause of yellow color of ‘Huangjinya’ may be due to the antioxidative activity of flavonoids. Solar UV-B radiation may cause serious damage including reduction of the photosynthetic fluorescence parameters, thylakoid electron transport, and photosynthetic pigments (chlorophyll and carotenes), and then induce lipoperoxidation in chloroplast membranes (Lidon and Ramalho, 2011). Activation of flavonoid biosynthesis exposed to light may be a response that protects tea leaves from the damages caused by sunlight-derived UV irradiation (Agati et al., 2012). However, the lower flavonoid content in ‘Huangjinya’ plants grown under sunlight make it difficult to resist UV-induced damages. Shading increased catechins and avoided UV-induced damage, and then made the leaf color turn green.

In addition to the well-known flavonoid synthesis-related genes, a novel gene *SRG1* was also mapped flavonoid biosynthesis in the present work. Although many *SRG1* genes have been released, their function is still unclear. The first report of *SRG1* showed that *SRG1* gene was expressed in senescing organs of Arabidopsis (Callard et al., 1996). Recently, the *SRG1* was found to have flavonoid-related functions in rice (Kim et al., 2013). In our study, the *SRG1* was mapped on the same position of *FLS* in KEGG pathway analysis. However, if *SRG1* also contribute to the catalysis of dihydroflavonols needs further confirmation. Moreover, *SRG1* expression profile was opposite to *FLS* but similar to that of *LDOX*, indicating that functional differentiation occurred in the oxoglutarate-dependent dioxygenase gene family in flavonoid biosynthesis.

### Carotenoid Biosynthesis is Responsible for ‘Huangjinya’ Color Changes

The first step in carotenoid biosynthesis is the production of phytoene from the condensation of geranylgeranyl diphosphate (GGPP) molecules, which are precursors of plastidial isoprenoids, including carotenoids (Bouvier et al., 2005; Ruiz-Sola et al., 2016). This biosynthetic process is catalyzed by phytoene synthase (PSY, **Figure 10**). Then, phytoene is sequentially desaturated and isomerized to produce phytofluene, 𝜀-carotene, and lycopene through desaturation reactions regulated by phytoene desaturase (PDS), ζ-carotene desaturase (ZDS), and ζ-carotene isomerase (ZISO), respectively. Furthermore, lycopene is cyclized to produce α-carotene and β-carotene, which are precursors of lutein (α-carotene) and zeaxanthin (β-carotene). In addition, carotenoids can be cleaved to produce apocarotenoids, including norisoprenoid, by carotenoid cleavage dioxygenases (CDDs) (Yahyaa et al., 2013; Hou et al., 2016).

The phenomenon that growing plants in the dark causes etiolation and that the green color could be recovered when the plants were returned to the light is previously known (Park et al., 2002; Tuan et al., 2013; Bou-Torrent et al., 2015). However, the opposite phenomenon appears to occur in ‘Huangjinya,’ which has yellow leaves when exposed to sunlight but green leaves when shaded. The etiolated leaves of ‘Huangjinya’ showed low carotenoid and total chlorophyll levels. However, both carotenoid and chlorophyll levels increased after shading (**Figure 1**). This observation is consistent with the expression patterns of carotenoid biosynthesis-related genes which were induced by shading treatment (**Figure 10**). Our result is similar to a previous study which found that down-regulation of *PSY* gene result in yellow leaves of *Oncidium* hybrid orchid (Liu et al., 2014). Silencing of *PSY* also impaired the chloroplast apparatus and chlorophyll level, and then reduce the photosynthetic efficiency (Liu et al., 2014). As well, the expression of carotenoid biosynthesis-related genes in this study is consistent with the study by Li et al. (2016) which showed that the expression of most carotenoid biosynthesis-related genes increased after moderate shading, and *PDS, ZDS*, lycopene 𝜀-cyclase (*LCYE*) increased significantly (*P* < 0.05). In ‘Huangjinya,’ carotenoid biosynthesis is light-sensitive and all of the carotenoid biosynthesis-related genes were down-regulated exposure to sunlight. These results support the notion that carotenoid biosynthesis in young leaves of ‘Huangjinya’ is inhibited by suppressing the expression of related genes under the light condition.

### Other Potential Mechanisms Involved in the Leaf Color Changes

In this study, some stress responsive genes were induced by the shading of ‘Huangjinya,’ especially heat shock proteins 70 and 90 (HSP70 and HSP90, **Figure 8**), which are cellular chaperones that participate in many cellular processes and are sensitive to heat stress. Chloroplast HSP90 could function within a chaperone complex that includes Tic110, Tic40, Toc75, Tic22, and stromal chaperones (HSP70 and HSP93) in the chloroplast stroma to promote membrane translocation during protein import into the chloroplast (Inoue et al., 2013). Additionally, the chloroplast-targeted HSP70 contributes to the molecular protection and repair of photosystem II during and after photoinhibition (Schroda et al., 1999). Stromal HSP70 has been found to interact with PDS in the chromoplasts of *Narcissus pseudonarcissus* (Bonk et al., 1996). Thus, shade-induced gene expression of these HSPs suggest potential roles in carotenoid biosynthesis in ‘Huangjinya.’

## Conclusion

Exposure to sunlight would inhibit the production of carotenoids and flavonoids in ‘Huangjinya’ by regulating flavonoid and carotenoid biosynthesis associated genes, and then result in the yellow color of ‘Huangjinya.’ Meanwhile, shading could reverse the process of flavonoid and carotenoid biosynthesis, and made the leaf color turn green. Thus, the leaf color changes in ‘Huangjinya’ are largely determined by the combined effects of flavonoid and carotenoid biosynthesis.

## Author Contributions

LS and XL designed the experiment. QM carried out most of the experiments and drafted manuscript. ZZ, KS, and YY did the data analyses. JT and NK revised and finalized the paper.

## Conflict of Interest Statement

The authors declare that the research was conducted in the absence of any commercial or financial relationships that could be construed as a potential conflict of interest.

## Footnotes

1 http://www.phrap.org/

2 http://www.genome.jp/tools/clustalw/

3 http://meme-suite.org/tools/meme

4 https://www.ncbi.nlm.nih.gov/geo/
