# Supplementary material for: Molecular Link between Leaf Coloration and Gene Expression of Flavonoid and Carotenoid Biosynthesis in Camellia sinensis Cultivar ‘Huangjinya’
Source: Front Plant Sci. 2017 May 24;8:803. doi: 10.3389/fpls.2017.00803 (PMC5443146; doi:10.3389/fpls.2017.00803)
Supplement: Supplementary file 1 [file Data_Sheet_1.docx]

Supplementary Material

Phenotype and transcriptome analysis reveals flavonoid and carotenoid biosynthesis together influence leaf colour formation in the light-sensitive albino Camellia sinensis cultivar ‘Huangjinya’

**Lubin Song^1＃^, Qingping Ma^2＃^, Zhongwei Zou ^3^, Kang Sun^2^, Yuantao Yao^1^, Jihan Tao^1^, Najeeb Ahmed^2^, Xinghui Li^2*^**

*** Correspondence:** Xinghui Li: lxh@njau.edu.cn

# 1 Supplementary Table

**Table S1** The primers used for genes on flavonoid and carotenoid syntheses.

| Gene ID | Annotation | Forward primer (5'-3') | Reverse primer (5'-3') |
| --- | --- | --- | --- |
| Flavonoid biosynthesis |  |  |  |
| CL1403.Contig2_All | Phenylalanine ammonia-lyase (PAL) | CAATAGGGAAGCTCATGTTTGC | CGCTTTGGACATGGTTGGTTAC |
| CL1270.Contig2_All | Cinnamate-4-hydroxylase (C4H) | TCACCGAGCCAGACACCTAC | CCTTAGCCTCCTCTTCCAAGA |
| CL1795.Contig2_All | Chalcone synthase (CHS) | GTGGGCCTTACATTTCATCTC | TCTAGTATGAATAGCACGCAC |
| CL5400.Contig2_All | Chalcone isomerase (CHI) | GTTAAGTGGAAGGGCAAGAC | GAAAGCAATCGTCAATGATCC |
| Unigene39726_All | Flavanone 3-hydroxylase (F3H) | TCTACCCGAAATGCCCACAAC | CCTCCCATTGCTTAGATAATG |
| Unigene28864_All | Flavonoid-3'-hydroxylase (F3'H) | TCGAATGGCATCTGACAGTTG | GCCTGCACCAAATGGTATGAC |
| Unigene8507_All | Flavonoid 3',5'-hydroxylase (F3'5'H) | GAGCACACGACGAGATGGAT | GTCTTTGCATTCTTTCCACTC |
| CL8169.Contig1_All | Flavonol synthase (FLS) | GCATGAGGTCAAGGAGGCTGT | GACAATCAGGGCATTAGGGATG |
| Unigene45768_All | Dihydroflavonol 4-reductase (DFR) | CACTAGGAATGAAGGACACTAC | GAACGACACAACTGGCAAGT |
| Unigene11476_All | Leucoanthocyanidin dioxygenase (LDOX) | CAGTAATCCGTGTTCAATCCTTG | TAAACCTGCTTCTCTTCCATG |
| Unigene7347_All | Leucoanthocyanidin reductase (LAR) | CTATGACAATACTCACCCATC | GAGTGCGTCCAATCTTCTTCT |
| CL11956.Contig1_All | Anthocyanidin reductase (ANR) | TCGAAAACACTAGCTGAGAAAG | GCTCGGGAACACTGGTATTG |
| Carotenoid biosynthesis |  |  |  |
| Unigene51424_All | Phytoene synthase (PSY) | GATAGAAGGAATGAGATTGGACC | TGTCCTCATCTGAAAGCCCTG |
| Unigene21827_All | Phytoene desaturase (PDS) | ATGGTAATGCTATTGAAGGAG | GTAATATTCCTTACATGCCAC |
| CL4803.Contig1_All | Zeta-carotene desaturase (ZDS) | CTGAAGACTATTACCTCGAGG | CTTCTGATCAGGTCTAAATGG |
| Unigene16394_All | Ζ-carotene isomerase (ZISO) | GTATCATGAGAATAACCAGGC | CACCATATCGTATAGCTAGCC |
| CL5617.Contig3_All | Lycopene ɛ-cyclase (LCYE) | GGATCATTACCGAATACAGAGC | GTTCCAAGCTAGCATCGAGAT |
| CL12353.Contig1_All | Lycopene β-cyclase (LCYB) | AGTTGTCGGCATTGGTGGTAC | GCTTAAGCAGAATATCCATACC |
| CL3692.Contig1_All | Carotene hydroxylase (CHY) | ATGCACGAGTCTCACCATAAAC | CTTCGGAGGTAGGGTACGTTG |
| CL2411.Contig1_All | Zeaxanthin epoxidase (ZEP) | CATAATAGATTCCAGTCCAGATG | CCATCTTTCGCTTCCATAAG |
| CL2738.Contig1_All | Violaxanthin de-epoxidase (VDE) | TCAGAGATTTGTGCAAGATCC | TCTAATTCGGGTACAATGCTG |
| Unigene19522_All | Neoxanthin synthase (NSY) | GGATGGTAGCGAGGTTGAGG | ATTCTTGTTGAGCCGAGGCAC |
| Unigene2350_All | Carotenoid cleavage dioxygenase 1 (CCD1) | GCCTTGAGAATCCAGATCTAGAC | GATGCTGTCCAATGTAGTTGC |
| CL11428.Contig2_All | CCD4 | GATGAGGTGGTTTGAAGTGCC | CACCCCACTCTTAAGATCAATC |
| CL1748.Contig2_All | CCD8 | GTGGGAAAGATTGTGGCGAG | TGCTGCCTCTAACTTCCCAG |

**2 Supplementary Figures**


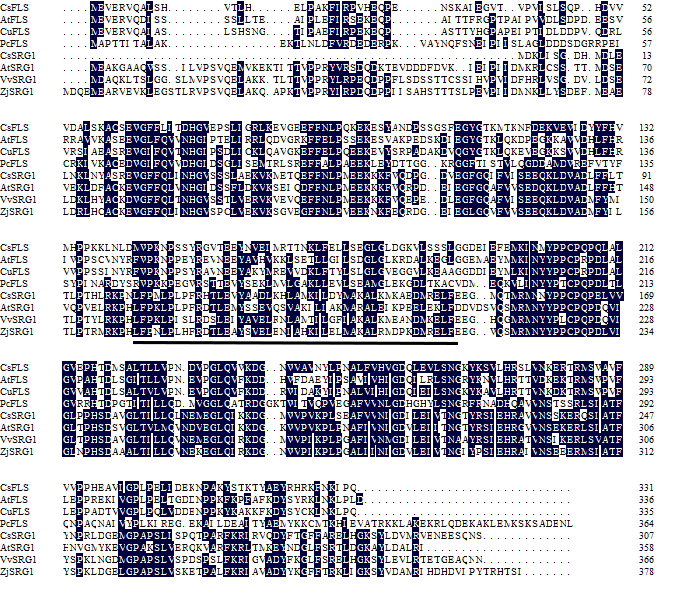


**Figure S1** Sequence alignment of SRG1 and FLS genes. Leu-enriched region was underlined. *Camellia sinensis* (CsSRG1, CL6306. Contig1; CsFLS, ABM88786.1), *Arabidopsis thaliana* (AtSRG1, Q39224.1; AtFLS, NP_001190266.1), *Citrus unshiu* (CuFLS, Q9ZWQ9.1), *Petroselinum crispum* (PcFLS, Q7XZQ8.1), *Vitis vinifera* (VvSRG1, XM_002269051.2), *Ziziphus jujuba* (ZjSRG1, XM_016021136.1).
